# HIV viral suppression in children and adolescents 2 years after transition to dolutegravir: a multicentre cohort study

**DOI:** 10.1097/QAD.0000000000003835

**Published:** 2024-01-10

**Authors:** Akash Devendra, Maurus Kohler, Motlatsi Letsika, Hape Khooa, Lipontso Motaboli, Malebanye Lerotholi, Nadine Tschumi, Niklaus D. Labhardt, Jennifer A. Brown

**Affiliations:** a Department of Pediatrics, Baylor College of Medicine; b Baylor International Pediatric AIDS Initiative, Houston, TX, USA; c Baylor College of Medicine Children's Foundation Lesotho, Maseru, Lesotho; d Division of Clinical Epidemiology, Department of Clinical Research, University Hospital Basel; e University of Basel, Basel, Switzerland; f SolidarMed, Partnerships for Health; g Ministry of Health of Lesotho, Maseru, Lesotho.

**Keywords:** dolutegravir, HIV, HIV integrase inhibitors, paediatrics, treatment outcome, viremia

## Abstract

**Background:**

Treatment failure is common among children and adolescents with HIV. Antiretroviral therapy (ART) containing dolutegravir has recently been rolled out across Africa, though long-term real-world data in paediatric populations are lacking. Here, we report treatment outcomes among children and adolescents in Lesotho who transitioned from nonnucleoside reverse transcriptase inhibitor (NNRTI)-based to dolutegravir-based ART through 2 years’ follow-up.

**Methods:**

Data were derived from two open cohort studies in Lesotho. Children and adolescents aged less than 18 years who transitioned from NNRTI-based to dolutegravir-based ART at least 18 months before data closure were included. We report viral load results less than 12 months before, 12 (window: 6–17) months after, and 24 (window: 18–29) months after transition to dolutegravir. Associations of pretransition demographic and clinical factors with 24-month viraemia were assessed through multivariable logistic regression.

**Results:**

Among 2126 included individuals, 1100 (51.7%) were female individuals, median age at transition to dolutegravir was 14.0 years [interquartile range (IQR) 11.5–15.8], and median time taking ART at transition was 7.6 years (IQR 4.4–10.6). Among those with a viral load result at the respective time points, viral suppression to less than 50 copies/ml was achieved by 1635 of 1973 (82.9%) less than 12 months before, 1846 of 2012 (91.8%) 12 months after, and 1725 of 1904 (90.6%) 24 months after transition to dolutegravir. Pretransition viraemia was associated with viraemia at 24 months, though more than 80% of individuals with pretransition viraemia achieved resuppression to less than 50 copies/ml at 24 months.

**Conclusion:**

The proportion of children and adolescents with viral suppression increased after transition to dolutegravir, though further progress is needed to reach global targets.

## Background

Since 2018, the World Health Organization (WHO) has recommended the integrase strand transfer inhibitor dolutegravir as the preferred core agent in antiretroviral therapy (ART) for most people with HIV, including children and adolescents [1]. Before this, the nonnucleoside reverse transcriptase inhibitors (NNRTIs) efavirenz and nevirapine were the preferred core agents for adults, adolescents, and older children starting ART, whilst children below 3 years of age were initiated on ART containing the protease inhibitor ritonavir-boosted lopinavir, or nevirapine [2,3]. Compared with previously preferred ART regimens, clinical trials have shown noninferior or superior efficacy of dolutegravir among adults [4–7] as well as children and adolescents [8]. Furthermore, dolutegravir is convenient, generally well tolerated, and unlike NNRTIs, has a high barrier to resistance [4,9].

Better options for paediatric ART were urgently needed: globally, in 2022, only 81% of children below 15 years of age and receiving ART achieved viral suppression below 1000 copies/ml, compared with 93% of adults [10]. In addition to social and psychological factors that may impede adherence to ART in these age groups [11], the lack of well tolerated, palatable, once-daily regimens with a high barrier to resistance has likely been contributory to lower proportions achieving viral suppression [12,13]. In some countries in sub-Saharan Africa, the prevalence of pretreatment resistance among infants aged up to 18 months who were newly diagnosed with HIV was more than 50% for NNRTIs and more than 10% for nucleoside reverse transcriptase inhibitors (NRTIs) [14]. Consequently, the rapidly progressing rollout of dolutegravir-based ART in low- and middle-income countries has held major promise for paediatric HIV care [15].

Real-world outcomes among adults appear promising [16–21]. However, observational studies describing paediatric treatment outcomes after the rollout of dolutegravir are currently scarce [18,22–25].

Lesotho, southern Africa, amended its guidelines introducing preferential use of dolutegravir-based ART in 2019 [26]. The major transition from NNRTI-based to dolutegravir-based ART, which was initially available for adolescents and for children weighing at least 20 kg, followed in 2020. More recently, dolutegravir has also become available for children of lower weight bands.

Here, we assess the programmatic transition from NNRTIs to dolutegravir among children and adolescents in care in Lesotho. This study aims to compare virologic treatment outcomes before and after transition to dolutegravir, and assess the association of pretransition viraemia, as well as other clinical and demographic factors, with posttransition treatment outcomes.

## Methods

### Study design and participants

This analysis includes routine clinical data from two cohorts: a routine care cohort maintained by Baylor College of Medicine Children's Foundation Lesotho (BCMCFL) comprising six BCMCFL clinics across the country (the Baylor Center of Excellence Maseru, and Satellite Centers of Excellence in Qacha's Nek, Mohale's Hoek, Leribe, Butha-Buthe, and Mokhotlong), and the prospective open Viral Load Cohort North-East Lesotho (VICONEL), which encompasses people receiving HIV care and viral load testing in Butha-Buthe and Mokhotlong districts and is maintained by the Division of Clinical Epidemiology, University Hospital Basel and the not-for-profit organisation SolidarMed.

For the present analysis, we included participants of these two cohorts who transitioned from an ART regimen consisting of an NNRTI (efavirenz or nevirapine) and two nucleoside reverse transcriptase inhibitors (NRTIs; lamivudine together with either abacavir, zidovudine, or tenofovir disoproxil fumarate) to a regimen consisting of dolutegravir and two NRTIs, and were less than 18 years old at the time of transition. Participants who had taken the NNRTI-based regimen for less than 6 months before transitioning or who transitioned less than 18 months before data closure (BCMCFL: 28 February 2023; VICONEL: 2 December 2023) were excluded.

### Setting

National guidelines in Lesotho call for 6-monthly viral load testing for children and adolescents, with enhanced adherence counselling and confirmatory viral load testing upon detection of viraemia [26–28]. Lesotho amended its national guidelines introducing dolutegravir as the preferred ART core agent in 2019, with most ART recipients transitioning to dolutegravir the following year [26]. The transition to dolutegravir occurred in a phased approach; people taking nevirapine-based ART, experiencing treatment failure or taking a nonfixed-dose combination of efavirenz-based ART were prioritized before those taking tenofovir disoproxil fumarate/lamivudine/efavirenz as a fixed-dose combination. From January 2020, a recent viral load or additional laboratory testing (notably creatinine clearance) were no longer required. For people with low-level viraemia (20–999 copies/ml), immediate transitioning with enhanced adherence counselling was recommended. For people with a viral load of at least 1000 copies/ml, adherence counselling and repeat viral load testing was recommended prior to transition. For people with persistent viraemia of at least 1000 copies/ml, optimisation of the NRTI backbone was recommended. HIV resistance testing was not part of routine care. As dolutegravir was initially only available as a fixed-dose combination (tenofovir disoproxil fumarate/lamivudine/dolutegravir) for people weighing at least 35 kg, and as a nonfixed-dose combination for people weighing at least 20 kg, onward treatment with lopinavir-based ART was recommended for children below 20 kg.

Participants in the BCMCFL cohort receive HIV care in physician-led, comparatively well equipped clinics run by BCMCFL in collaboration with the Lesotho Ministry of Health. The urban BCMCFL Center of Excellence in Maseru, contributing over half the participants in the BCMCFL cohort, is the largest paediatric HIV clinic in Lesotho and comprises a multidisciplinary staff to provide clinical care, medication and psychosocial support to children and adolescents with HIV as well as their families.

VICONEL has been described in more detail elsewhere [29,30]. The present analysis includes VICONEL participants from 3 hospitals and 18 health centres run by the Ministry of Health or the Christian Health Association of Lesotho. These facilities are predominantly rural and nurse-led.

### Data collection, measures, and statistical analysis

#### Data collection

BCMCFL clinics utilize standardized electronic medical records for each clinical encounter. Abstraction scripts were generated to pull required data into spreadsheets. In VICONEL, each viral load test triggers a data entry, whereas clinic visits are not captured. VICONEL includes data from all viral load testing conducted at Butha-Buthe Government Hospital, which provides viral load testing for Butha-Buthe and Mokhotlong districts, and from additionally available point-of-care viral load testing at three facilities. Results are entered into the secured database alongside associated demographic and treatment data.

As two BCMCFL Satellite Centers of Excellence fall within the VICONEL catchment area, and considering the possibility of mobility between the cohorts, we merged datasets on the basis of participants’ national ART numbers. In case of overlap, data from BCMCFL was used.

#### Measures

We report demographic, clinical, and treatment history data at the time of transition to dolutegravir-based ART, stratified by the pretransition viral load (<12 months before transition). Viral load results are reported at less than 12 months before transition to dolutegravir (if multiple results were available within this time frame, the most recent was considered), at 12 (window: 6–17 months; closest viral load to 12 months considered) and 24 months (window: 18–29 months; closest viral load to 24 months considered) after transition. We only considered viral loads for the 12-month window if taken at least 6 months before the viral load in the 24-month window. We further report the last available viral load result both before and after transition to dolutegravir, regardless of time windows. Viral load results were categorized as viral suppression (<50 copies/ml), low-level viraemia (50–999 copies/ml), or virological failure (≥1000 copies/ml). In BCMCFL, adherence was assessed through pill count (total pills dispensed at the previous visit subtracting the number of returned pills, divided by the number of days since the last dispense) and categorized as adherent (pill count 95–105%) vs. nonadherent (pill count <95 or >105%). VICONEL does not collect pill count data. Classification of immunodeficiency at diagnosis followed the Lesotho National ART guidelines [28], using CD4^+^ count or, where available for participants aged less than 5 years of age, CD4^+^ percentages. Participants were followed up until the last viral load measurement available or until loss to follow-up, transfer out, or death.

#### Statistical analysis

Categorical variables were described as frequency and percentage, continuous variables as median and interquartile ranges. Among participants with viral load results at all three time points, a Sankey plot was used to visualize viral load dynamics. We used a logistic regression model to estimate the association of exposure variables with viraemia (≥50 copies/ml) 24 months after transition. Exposure variables were the viral load category of the pretransition viral load (<12 months before transition, categorized as: missing viral load; <50 copies/ml; 50–999 copies/ml; ≥1000 copies/ml), sex (male; female); age at transition (<12 years; ≥12 years), NNRTI core agent before transition (efavirenz; nevirapine), and cohort (BCMCFL; VICONEL). Sensitivity and further regression analyses were performed for a different threshold of viraemia (≥1000 copies/ml), being lost to follow-up or having an unknown outcome status at 24 months, and adding pill count data at 24-month viral load as an exposure variable (available for BCMCFL only). McNemar tests were used to assess changes of proportions in adhering vs. nonadhering participants at less than 12 months before transition, 12-month and 24-month post transition. All analyses were conducted using STATA version 16.1.

### Ethical considerations

The National Health Research Ethics Committee of Lesotho (NH-REC) and the Baylor College of Medicine institutional review board have approved data extraction and analysis from routinely collected participant-level data for people attending BCMCFL sites (ID 19-2014 and H34569, respectively). The VICONEL cohort was approved by NH-REC (ID134-2016). For both cohorts, consent was waived for the analysis of routine observational data.

## Results

### Participant characteristics

**Fig. 1:**
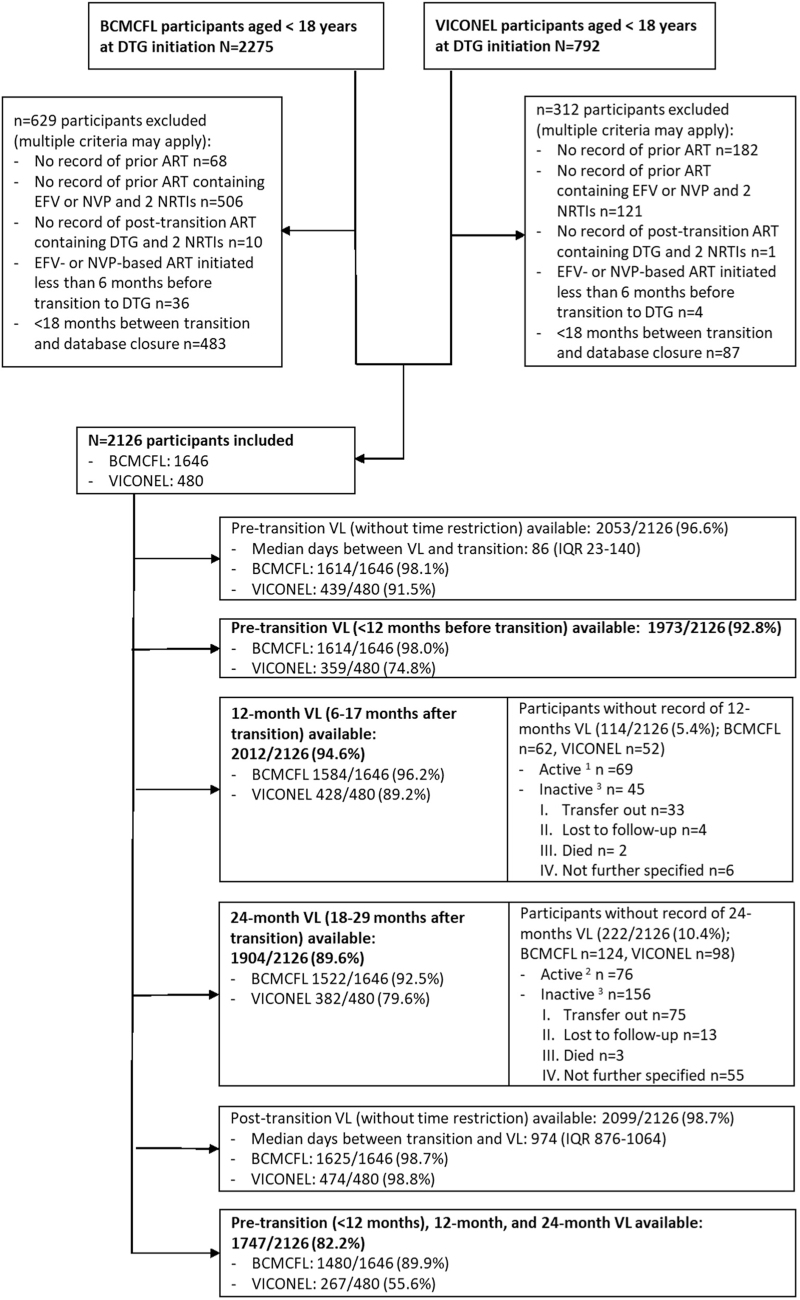
Study flow chart.

ART, antiretroviral therapy; BCMCFL, Baylor College of Medicine Children's Foundation Lesotho; DTG, dolutegravir; EFV, efavirenz; IQR, interquartile range; NRTI, nucleoside reverse transcriptase inhibitor; NVP, nevirapine; VICONEL, Viral Load Cohort North-East Lesotho; VL, viral load.

Among 3067 individuals (2275 BCMCFL; 792 VICONEL) aged less than 18 years at initiation of a dolutegravir-based ART regimen, 941 (629 BCMCFL; 312 VICONEL) were excluded as they did not meet all inclusion criteria (Figure 1). The remaining 2126 (1646 BCMCFL; 480 VICONEL) were included in this analysis. Around half of participants were female (1100/2122, 51.7%), median age at transition to dolutegravir was 14.0 (IQR 11.5–15.8) years, and median time on ART at transition was 7.6 (IQR 4.4–10.6) years (Table 1). Most participants (1438/2126, 67.6%), were taking an efavirenz-based ART regimen before transition; the remainder were taking nevirapine-based ART. Tenofovir disoproxil fumarate/lamivudine/dolutegravir was the most used regimen after transition (1133/2126, 53.3%; Table 1; see Supplementary Table 1 for backbone changes after transition to dolutegravir). Participants were followed up for a median of 2.7 (IQR 2.4–2.9) years after transition.

**Table 1:** Participant characteristics.

	Total (*n* = 2126)	VL result <12 months before transition to DTG			
		<50 copies/ml (*n* = 1635)	50–999 copies/ml (*n* = 204)	≥1000 copies/ml (*n* = 134)	Missing (*n* = 153)
Cohort					
BCMCFL	1646 (77.4)	1402 (85.7)	128 (62.7)	84 (62.7)	32 (20.9)
VICONEL	480 (22.6)	233 (14.3)	76 (37.3)	50 (37.3)	121 (79.1)
Facility type [*n* (%)]^a^					
BCMCFL Center of Excellence (Maseru)	869 (40.9)	773 (47.3)	54 (26.5)	26 (19.4)	16 (10.6)
BCMCFL Satellite Center of Excellence	777 (36.6)	629 (38.5)	74 (36.3)	58 (43.3)	16 (10.6)
Hospital within VICONEL	79 (3.7)	39 (2.4)	9 (4.4)	7 (5.2)	24 (15.9)
Nurse-led peripheral clinic within VICONEL	399 (18.8)	194 (11.9)	67 (32.8)	43 (32.1)	95 (62.9)
Female [*n* (%)]	1100 (51.7)	852 (52.1)	104 (51.0)	59 (44.0)	85 (55.6)
Age in years at transition to DTG [median (IQR)]	14.0 (11.5–15.8)	14.0 (11.7–15.8)	14.1 (11.3–16.1)	14.2 (10.8–16.4)	13.6 (11.1–15.8)
Weight at transition (kg) [median (IQR)]^b^	16 (11–23)	16 (11–23)	18 (13–23)	17 (14–26)	17.5 (10–24)
Height at transition (cm) [median (IQR)]^c^	106 (84–123)	105 (82–123)	110 (93–129)	114 (94–125)	106.5 (85–121)
Years between HIV diagnosis and transition to DTG [median (IQR)]^d^	9.6 (6.1–11.8)	9.7 (6.2–11.8)	8.6 (4.9–11.4)	9.5 (6.6–11.8)	8.9 (6.6–11.9)
Years on ART at transition to DTG [median (IQR)]	7.6 (4.4–10.6)	7.9 (4.4–10.8)	6.6 (3.8–10.1)	7.1 (5.4–10.2)	6.7 (3.7–9.8)
Classification of immunodeficiency at diagnosis [*n* (%)]^e^					
Not significant	584 (35.8)	414 (33.0)	68 (41.2)	44 (40.7)	58 (56.3)
Mild	168 (10.3)	125 (10.0)	24 (14.5)	12 (11.1)	7 (6.8)
Advanced	317 (19.4)	264 (21.0)	21 (12.7)	14 (13.0)	18 (17.5)
Severe	562 (34.5)	452 (36.0)	52 (31.5)	38 (35.2)	20 (19.4)
Years of follow-up after transition to DTG, median (IQR)^f^	2.7 (2.4–2.9)	2.7 (2.4–2.9)	2.7 (2.4–3.0)	2.7 (2.1–3.0)	2.9 (2.2–3.4)
Last regimen before transition to DTG [*n* (%)]					
ABC/3TC/EFV	716 (33.7)	547 (33.5)	83 (40.7)	32 (23.9)	54 (35.3)
AZT/3TC/EFV	613 (28.8)	489 (29.9)	55 (27.0)	34 (25.4)	35 (22.9)
TDF/3TC/EFV	109 (5.1)	76 (4.6)	13 (6.4)	6 (4.5)	14 (9.2)
ABC/3TC/NVP	23 (1.1)	20 (1.2)	0 (0.0)	2 (1.5)	1 (0.7)
AZT/3TC/NVP	665 (31.3)	503 (30.8)	52 (25.5)	60 (44.8)	49 (32.0)
First regimen after transition to DTG [*n* (%)]^g^					
ABC/3TC/DTG	730 (34.3)	541 (33.1)	81 (39.7)	45 (33.6)	63 (41.2)
AZT/3TC/DTG	263 (12.4)	213 (13.0)	16 (7.8)	26 (19.4)	8 (5.2)
TDF/3TC/DTG	1133 (53.3)	881 (53.9)	107 (52.5)	63 (47.0)	82 (53.6)

3TC, lamivudine; ABC, abacavir; ART, antiretroviral therapy; AZT, zidovudine; BCMCFL, Baylor College of Medicine Children's Foundation Lesotho; DTG, dolutegravir; EFV, efavirenz; IQR, interquartile range; NVP, nevirapine; TDF, tenofovir disoproxil fumarate; VICONEL, Viral Load Cohort North-East Lesotho; VL, viral load.

a Missing for 2 of 2126 (0.1%).

b Not available in VICONEL; overall missing for 1042 of 2126 (49.0%).

c Not available in VICONEL; overall missing for 1050 of 2126 (49.4%).

d Not available in VICONEL; overall missing for 569 of 2126 (26.8%).

e Missing for 495 of 2126 (23.3%).

f Missing for 27 of 2126 (1.3%) participants without any viral load after transition to dolutegravir.

g Three hundred and fifteen of 2126 (14.8%) had a backbone change after a median of 265 (IQR 136–429) days after initiation of dolutegravir (see Supplementary Table 1 for backbone changes).

### Treatment outcomes

Out of 2126 included participants, 1973 (92.8%) had an available viral load result less than 12 months prior to transition from NNRTI- to dolutegravir-based ART; 2012 of 2126 (94.6%) had at least one available viral load 12 months after transition; 1904 of 2126 (89.6%) participants had a viral load result 24 months after transition; and 1747 of 2126 (82.2%) had a viral load at all three time points (Fig. 1). Among those without viral load data at 24 months, 76 of 222 (34.2%) were active in care, 75 of 222 (33.8%) were reported as having transferred out, 13 of 222 (5.9%) were lost to follow-up, 3 of 222 (1.4%) died, and 55 of 222 (24.8%; BCMCFL 19, VICONEL 36) were inactive without further information available. Among the three participants who died, two deaths were directly attributed to HIV/AIDS; both had a history of poor adherence, virological and clinical failure both before and after transition to dolutegravir. The third participant had severe chronic lung disease, malnutrition, and a history of poor adherence but achieved viral resuppression after transitioning to dolutegravir. This death was attributed to complications of the chronic lung disease.

Regardless of viral load windows, 2053 of 2126 (96.6%) had a viral load available at any time while taking efavirenz-based or nevirapine-based ART before transition to dolutegravir (Table 2). Of these, at the last available viral load before transition, 1682 of 2053 (81.9%) showed viral suppression, 214 of 2053 (10.4%) had low-level viraemia, and 157 of 2053 (7.6%) virological failure. A viral load any time after transition was available for 2099 of 2126 (98.7%) participants. Of these, at the last available viral load after transition, 1875 of 2099 (89.3%), 139 of 2099 (6.6%), and 85 of 2099 (4.0%), respectively, had viral suppression, low-level viraemia, or virological failure. Viral suppression dynamics between the last-available viral load before and after transition are shown in Supplementary Figure 1.

**Table 2:** Treatment outcomes and adherence.

	Total	VL result <12 months before transition to DTG			
	(*n* = 2126)	<50 copies/ml (*n* = 1635)	50–999 copies/ml (*n* = 204)	≥1000 copies/ml (*n* = 134)	Missing (*n* = 153)
Days between pretransition VL (<12 months before transition; stratification factor) and transition, median (IQR)	84 (14–126)	84 (0–120)	90 (36–148)	84 (49–134)	NA
Last available VL before transition [*n* (%)]^a^					
<50 copies/ml	1682 (81.9)	1635 (100)	0 (0)	0 (0)	47 (58.8)
50–999 copies/ml	214 (10.4)	0 (0)	204 (100)	0 (0)	10 (12.5)
≥1000 copies/ml	157 (7.6)	0 (0)	0 (0)	134 (100)	23 (28.s)
Days from last available VL before transition to transition, median (IQR)^a^	86 (23–140)	84 (0–120)	90 (36–148)	84 (49–134)	525 (423.5–661)
12-month VL [*n* (%)]^b^					
<50 copies/ml	1846 (91.7)	1472 (94.2)	162 (84.4)	104 (81.9)	108 (82.4)
50–999 copies/ml	117 (5.8)	64 (4.1)	24 (12.5)	15 (11.8)	14 (10.7)
≥ 1000 copies/ml	49 (2.4)	26 (1.7)	6 (3.1)	8 (6.3)	9 (6.9)
Days from transition to 12-month VL [median (IQR)]^b^	363 (309–408.5)	363 (309–406)	362 (309–418)	365 (336–412)	351 (292–425)
24-month VL [*n* (%)]^c^					
<50 copies/ml	1725 (90.6)	1379 (92.0)	150 (83.3)	99 (87.6)	97 (86.6)
50–999 copies/ml	104 (5.5)	72 (4.8)	19 (10.6)	5 (4.4)	8 (7.1)
≥1000 copies/ml	75 (3.9)	48 (3.2)	11 (6.1)	9 (8.0)	7 (6.3)
Days from transition to 24-month VL [median (IQR)]^c^	727 (673–778)	728 (673–778)	719 (661–776)	725 (686–784)	736.5 (662.5–790)
Last available VL after transition [*n* (%)]^d^					
<50 copies/ml	1875 (89.3)	1478 (91.4)	162 (80.2)	109 (82.0)	126 (85.7)
50–999 copies/ml	139 (6.6)	89 (5.5)	29 (14.4)	13 (9.8)	8 (5.4)
≥ 1000 copies/ml	85 (4.0)	50 (3.1)	11 (5.4)	11 (8.3)	13 (8.8)
Days from transition to last available VL after transition [median (IQR)]^d^	974 (876.5–1064)	972 (883–1048)	984.5 (867–1105)	974 (784–1108)	1057 (814–1248)
Adherent at pretransition VL (<12 months before transition) [*n* (%)]^e^	*n* = 1273 1119 (87.9)	*n* = 1096 966 (88.1)	*n* = 109 97 (89.0)	*n* = 68 56 (82.4)	NA
Adherent at 12-months VL [*n* (%)]^f^	*n* = 1159 1010 (87.1)	*n* = 997 864 (86.7)	*n* = 8677 (89.5)	*n* = 59 52 (88.1)	*n* = 17 17 (100.0)
Adherent at 24-months VL [*n* (%)]^g^	*n* = 928 789 (85.0)	*n* = 812 689 (84.9)	*n* = 6153 (86.9)	*n* = 45 40 (88.9)	*n* = 10 7 (70.0)
Adherence pre and 24 months after transition in BCMCFL [*n* (%)]	*n* = 721	*n* = 636	*n* = 50	*n* = 35	
Adherent pretransition and posttransition	553 (76.7)	489 (76.9)	38 (76.0%)	26 (74.3)	NA
Nonadherent pretransition and adherent posttransition	64 (8.9)	54 (8.5)	4 (8.0%)	6 (17.1)	NA
Adherent pretransition and nonadherent posttransition	86 (11.9)	78 (12.3)	6 (12.0%)	2 (5.7)	NA
Nonadherent pretransition and posttransition	18 (2.5)	15 (2.4)	2 (4.0%)	1 (2.9)	NA

BCMCFL, Baylor College of Medicine Children's Foundation Lesotho; IQR, interquartile range; VL, viral load.

a Last available viral load pretransition missing for 73 of 2126 (3.4%).

b Twelve-month viral load missing for 114 of 2126 (5.4%); see Fig. 1 for participants’ status in case of missing 12-month viral load.

c Twenty-four-month viral load missing for 222 of 2126 (10.4%); see Fig. 1 for participants’ status in case of missing 24-month viral load.

d Last available viral load posttransition missing for 27 of 2126 (1.3%).

e Not available in VICONEL. Among BCMCFL cohort participants with a pretransition viral load, adherence data missing 341 of 1614 (21.1%).

f Not available in VICONEL. Among BCMCFL cohort participants with a 12-month viral load, adherence data missing 425 of 1584 (26.8%).

g Not available in VICONEL. Among BCMCFL cohort participants with a 24-month viral load, adherence data missing 594 of 1522 (39%).

**Fig. 2:**
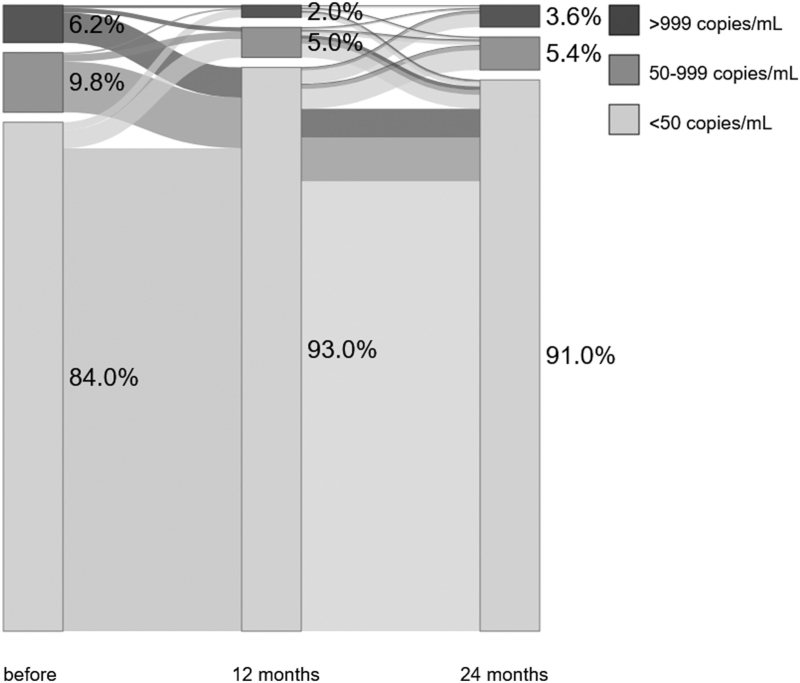
Viral load dynamics of participants with an available viral load less than 12 months pretransition, 12 months after transition, and 24 months after transition to dolutegravir (*n* = 1747).

At the pretransition viral load (<12 months before transition), 1635 of 1973 (82.9%) participants had viral suppression, 204 of 1973 (10.3%) low-level viraemia and 134 of 1973 (6.8%) virological failure, respectively. At the 12-month viral load, 1846 of 2012 (91.7%) participants had viral suppression, 117 of 2012 (5.8%) low-level viraemia and 49 of 2012 (2.4%) virological failure, respectively. At the 24-month viral load, 1725 of 1904 (90.6%) participants had viral suppression, 104 of 1904 (5.5%) low-level viraemia and 75 of 1904 (3.9%) virological failure, respectively. Out of 108 participants with virological failure pretransition (<12 months before transition) and available viral load results at 12 and 24 months, 87 (80.6%) and 97 (89.8%) were virally suppressed at 12 and 24 months, respectively. Viral load dynamics of participants with viral loads for all three time points (<12 months pretransition, 12-month and 24-month viral load, *n* = 1747) are shown in Fig. 2. Most participants were virally suppressed at all three time points (1297/1747, 74.2%).

The shades of nodes indicate viral load categories at the respective time point. The shades of flows indicate the pretransition viral load category.

Pill count data were available for the BCMCFL cohort. Among individuals with available pill count data at the respective time point, 1119 of 1273 (87.9%) had a record of being adherent less than 12 months before, 1010 of 1159 (87.1%) 12 months after, and 789 of 928 (85.0%) 24 months after transition to dolutegravir (Table 2). Documented adherence did not change significantly between the viral loads pre-transition and 12 months after transition (chi^2^ = 0.01, *P* value = 0.94) or between the viral loads pretransition and 24 months after transition (chi^2^ = 3.23, *P* value = 0.07). Most participants in the BCMCFL cohort had documented adherence at both the pretransition viral load and the 12-month viral load (703/914, 76.9%), as well as both the pretransition viral load and the 24-month viral load (553/721, 76.7%).

### Factors associated with 24-month viraemia

**Fig. 3:**
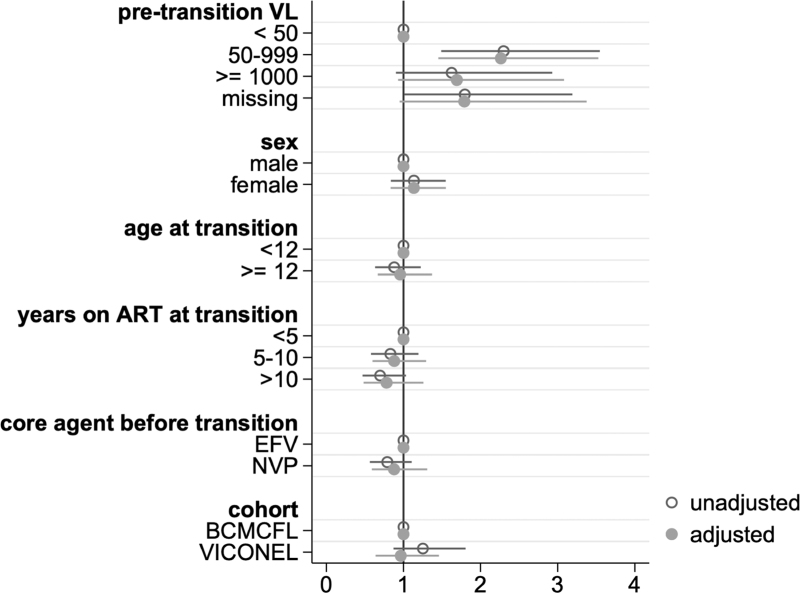
Unadjusted and adjusted odds ratios for viraemia (≥50 copies/ml) 24 months after transition to dolutegravir (*n* = 1904).

ART, antiretroviral therapy; BCMCFL, Baylor College of Medicine Children's Foundation Lesotho; EFV, efavirenz; NVP, nevirapine; VICONEL, Viral Load Cohort North-East Lesotho; VL, viral load.

In a multivariable logistic regression model, participants with a pretransition viral load (<12 months before transition) of 50–999 copies/ml had higher odds of a 24-months viral load of at least 50 copies/ml [aOR (95% CI) 2.3 [1.5–3.5]; Fig. 3) than those with pretransition viral suppression. Pretransition viral loads (<12 months before transition) that were at least 1000 copies/ml or missing were not clearly associated with viraemia at 24 months; however, confidence intervals were wide [1.7 (0.9–3.1); 1.8 (0.9–3.3, respectively)]. Sex, age at transition, time on ART before transition, ART core agent before transition, and cohort also did not show a statistically significant association with viraemia at 24 months. Inclusion of the 24-month ART backbone in the regression model led to similar results, with no association between the backbone and 24-month viraemia (data not shown). Results from sensitivity and further regression analyses are shown in Supplementary Figure 2. A model using a higher threshold of viraemia (≥1000 copies/ml) showed similar results, though here, a pretransition viral load (<12 months before transition) of at least 1000 copies/ml rather than 50–999 copies/ml was associated with 24-month viraemia. In an analysis where being lost-to follow-up or having an unknown status at 24 months was the outcome of interest, odds were higher for VICONEL compared with BCMCFL participants [aOR 2.7 (1.6–4.8)], but lower with increasing time on ART at transition. Finally, within BCMCFL, no clear association of 24-month adherence assessed by pill count and 24-month viraemia was observed.

## Discussion

In this study, we assessed virological outcomes of children and adolescents with HIV transitioning from NNRTI-based to dolutegravir-based ART in Lesotho. To our knowledge, this is the first study reporting 2-year outcomes upon programmatic transition to dolutegravir in a paediatric population in sub-Saharan Africa.

Our results showed encouraging viral suppression rates 1 and 2 years after transition. Over 95% of participants with a 2-year viral load had a result less than 1000 copies/ml. However, when applying a threshold of 50 copies/ml, only 91.7% and 90.6% were virally suppressed 1 and 2 years after transition, respectively. Importantly, the vast majority of participants with pretransition viraemia became virally suppressed after transition to dolutegravir and maintained suppression throughout the 2-year follow-up.

Unsurprisingly, we observed an association of pre-transition viraemia with 24-month viraemia at various viral load thresholds, supporting the 2021 WHO guidelines lowering the threshold for viral suppression [31]. Our findings did not reveal a statistically significant correlation between a viral load at least 1000 copies/ml pretransition or the absence of a pretransition viral load and 24-month viraemia. Nonetheless, it is plausible that a larger sample size could yield a significant effect. The proportion with a missing viral load at any time point and the odds of being lost to follow-up or having an unspecified status at 24 months were higher in VICONEL than in BCMCFL. This is likely in part an artefact of cohort setup and the points of data entry into the cohort (BCMCFL: clinic visit; VICONEL: viral load measurement) but may also be indicative of better follow-up in relatively well resourced, physician-led BCMCFL facilities compared with primarily nurse-led, resource-constrained facilities in VICONEL. Yet, VICONEL participants with 24-month viral load results did not have a higher odds of viraemia after transitioning to dolutegravir compared with BCMCFL participants.

This study comes with limitations. First, the 24-month viral load was missing for 222 (10.4%) participants, including 68 (3.2%) classified as either lost to follow-up or not further specified. This number likely includes a substantial number who remained in care but transferred out of the respective cohort, especially within VICONEL, which does not have a system to capture transfers to non-VICONEL clinics. Nevertheless, children and adolescents with HIV with missing viral loads might have higher rates of viraemia and subsequent poor outcomes, as described in a multinational tracing study [32] for the predolutegravir era. Second, there may be unmeasured confounders. Of note, temporal effects as well as the effect of increasing participant age, for example, on adherence, are not corrected for. Third, no resistance data were available. Lastly, we did not assess the effect of backbone changes at transition on viraemia at 24 months in this paediatric population, as in growing children, backbone changes are expected to occur independently of viral load for regimen simplification, notably changes to tenofovir disoproxil fumarate/lamivudine available for individuals weighing at least 35 kg [28]. At the time of the study, tenofovir disoproxil fumarate/lamivudine was the only backbone available as a fixed-dose combination first with efavirenz and later with dolutegravir for individuals weighing at least 35 kg [28,31]. Thus, the increase from 5.1% before to 53.3% after transition to DTG who had tenofovir disoproxil fumarate/lamivudine as a backbone may have contributed to the improved virological outcomes at 24 months; however, we did not observe any association of the 24-month NRTI backbone with 24-month viraemia.

Our findings of viral suppression rates are in line with some studies among paediatric populations in sub-Saharan Africa assessing virological outcomes up to 1 year after transition to dolutegravir. In studies in Kenya and at Baylor sites in Botswana, Eswatini, Lesotho, Malawi, Tanzania, and Uganda [23,33], the authors reported viral suppression rates of 93.4–95.8% when applying a threshold of 1000 copies/ml, where in our study, 96.1% had a viral load less than 1000 copies/ml at 24 months. However, other studies have reported lower viral suppression rates after transition to dolutegravir (Mozambique: 76.2% [25]; Tanzania: 82.5% [34]), raising questions about the comparability of HIV programs and its populations in sub-Saharan Africa [35].

Compared with the paediatric data presented here, a study reporting 2-year outcomes of adult participants in VICONEL reported higher rates of viral suppression for both thresholds (50 copies/ml: 95.1%; 1000 copies/ml: 99.1%) [21], again highlighting the discrepancy between treatment outcomes of paediatric and adult populations. The observed association of pretransition viraemia with viraemia after transition to dolutegravir is consistent with studies conducted in adult populations [16,17], as are the reassuring viral load dynamics of participants with pretransition viraemia who mostly achieved viral suppression after transition [16,19,21]. Lastly, assessing adherence data of BCMCFL, we did not observe a significant association of nonadherence with viraemia at 24 months. This is in line with recent studies reporting challenges in predicting virological outcomes with records of adherence [36,37].

In conclusion, real-world longitudinal outcomes of children and adolescents who transitioned from NNRTI-based to dolutegravir-based ART are encouraging. Nevertheless, the fraction of participants with viraemia greater than 50 copies/ml 2 years after transition remains high, necessitating substantial further improvements in care for these age groups.

## Acknowledgements

We thank the staff at BCMCFL clinics for contributions to the BCMCFL cohort. We thank diagnostic and data personnel at Butha-Buthe Government Hospital and SolidarMed, notably Katleho Tlali and Moliehi Mokete, for their contributions to VICONEL. Finally, we gratefully acknowledge the participants.

Author contributions: Mo.L., A.D., M.K., N.D.L., and J.A.B. conceived and/or designed the analysis. A.D., M.o.L., and H.K. contribute to the implementation of the BCMCFL cohort. A.D., Mo.L., and H.K., extracted and prepared data from the BCMCFL cohort. L.M., Ma.L., N.T., N.D.L., and J.A.B. contribute to implementation of VICONEL. L.M., Ma.L., N.T., J.A.B., and M.K. extracted and prepared data from VICONEL. M.K. merged the datasets and performed the analyses, with key input from A.D., J.A.B., N.T., and N.D.L. A.D., M.K., and J.A.B. wrote the first draft of the manuscript. All authors contributed to and approved the final manuscript.

Funding: BCMCFL clinical sites receive core funding from the Ministry of Health Lesotho, Government of Lesotho. VICONEL has been funded by the Swiss National Science Foundation (IZ07Z0_160876/1, obtained by N.D.L.; PCEFP3_181355, obtained by N.D.L.) and ESTHER Switzerland (obtained by N.D.L.). M.K. receives his salary through a grant from Janggen-Pöhn Foundation (obtained by M.K.). J.A.B. receives her salary through grants from Fondation Botnar (REG-19-008, obtained by N.D.L. and J.A.B.) and the University of Basel Research Fund Junior Researchers (3ZX1422, obtained by J.A.B.).

### Conflicts of interest

N. D. L. reports having received travel grants to attend scientific conferences from Gilead Sciences and ViiV Healthcare; in 2022 and 2023, his division received honoraria from ViiV Healthcare, the Research Council of Norway, and the Swiss National Science Foundation; he also reports participation on a data and safety monitoring board for a phase 2 trial of Pharming (compensation to division, not author). All other authors report no potential conflicts.

## Supplementary Material

Supplemental Digital Content
