# Supplementary material for: HIV viral suppression in children and adolescents 2 years after transition to dolutegravir: a multicentre cohort study
Source: AIDS. 2024 Jan 10;38(7):1013–23. doi: 10.1097/QAD.0000000000003835 (PMC11064912; doi:10.1097/QAD.0000000000003835)
Supplement: Supplemental Digital Content [file aids-38-1013-s001.docx]

# Supplementary Material

Section A: Viral load dynamics

Section B: Regimen changes between transition and the 24-month viral load

Section C: Further regression analyses

Section A: Viral load dynamics


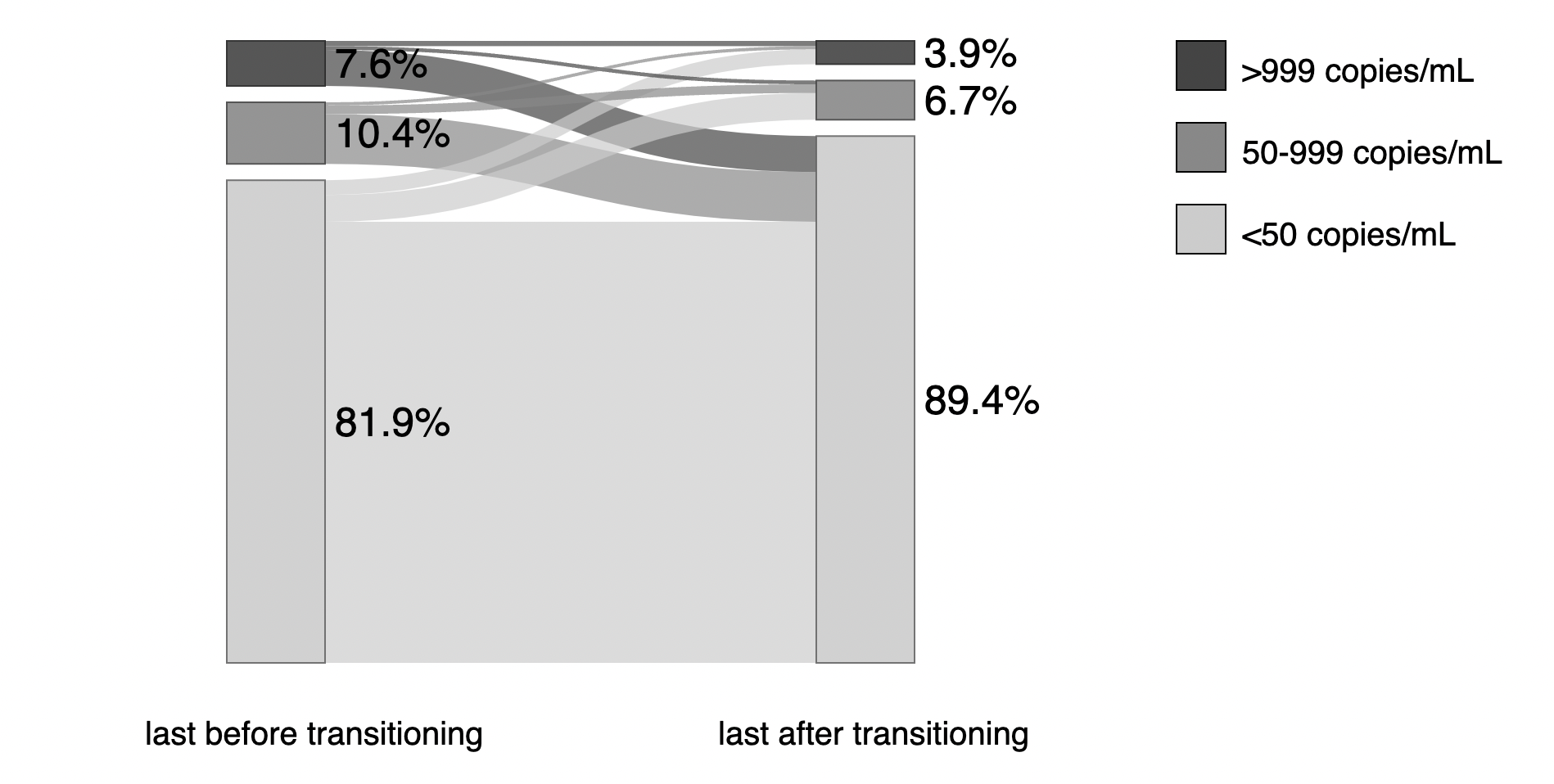


Supplementary Figure 1: Viral load dynamics of participants with at least one viral load before and one viral load after transition (n=2031). The median time between the last viral load available before transitioning and transition was 86 (IQR 23-140) days. The median time between transition and the last viral load available after transition were 974 (IQR 876.5 – 1064) days. The proportion for the viral load categories at the respective time point is indicated by the shades of the nodes. The shades of flows indicate the pre-transition viral load category.

Section B: Regimen changes between transition and the 24-month viral load

Supplementary Table 1: Type of regimen change. No discontinuations of DTG were reported.

| First regimen after transitioning | Second regimen after transitioning | Frequency |
| --- | --- | --- |
| ABC-3TC-DTG | TDF-3TC-DTG | 156 |
| AZT-3TC-DTG | ABC-3TC-DTG | 87 |
| AZT-3TC-DTG | TDF-3TC-DTG | 67 |
| TDF-3TC-DTG | ABC-3TC-DTG | 2 |
| ABC-3TC-DTG | AZT-3TC-DTG | 2 |
| TDF-3TC-DTG | AZT-3TC-DTG | 1 |

Abbreviations: ABC, abacavir; AZT, zidovudine; TDF, tenofovir disoproxil fumarate; 3TC, lamivudine; EFV, efavirenz; NVP, nevirapine; DTG, dolutegravir

Section C: Further regression analyses

Supplementary Figure 2: Unadjusted and adjusted odds ratios for **(A)** virological failure (≥ 1000 copies/mL) at the 24-month VL (n=1904); **(B)** being classified as lost to follow-up or having an unknown outcome status at the 24-month time point (n=2126); and **(C)** viraemia (≥ 50 copies/mL) at 24-month VL after transition, additionally adjusted for pill count data at 24-month VL (n=928; only available for BCMCFL).
